# Observed management practices in relation to the risk of infection with paratuberculosis and to the spread of *Mycobacterium avium* subsp. *paratuberculosis* in Swiss dairy and beef herds

**DOI:** 10.1186/1746-6148-10-132

**Published:** 2014-06-14

**Authors:** Rahel Künzler, Paul Torgerson, Selina Keller, Max Wittenbrink, Roger Stephan, Gabriela Knubben-Schweizer, Beat Berchtold, Mireille Meylan

**Affiliations:** 1 Clinic for Ruminants, Vetsuisse Faculty, University of Berne, Bremgartenstrasse 109a, 3012 Berne, Switzerland; 2 Section of Veterinary Epidemiology, Vetsuisse Faculty, University of Zurich, Zurich, Switzerland; 3 Institute of Veterinary Bacteriology, Vetsuisse Faculty, University of Zurich, Zurich, Switzerland; 4 Institute for Food Safety and Hygiene, Vetsuisse Faculty, University of Zurich, Zurich, Switzerland; 5 Clinic for Ruminants with Ambulatory and Herd Health Services at the Centre for Clinical Veterinary Medicine, Ludwig-Maximilians-University, Munich, Germany

**Keywords:** Paratuberculosis, Johne’s disease, *Mycobacterium avium* ssp. *paratuberculosis*, Infection, Awareness, Risk factor, Control, Dairy, Beef

## Abstract

**Background:**

Many studies have been conducted to define risk factors for the transmission of bovine paratuberculosis, mostly in countries with large herds. Little is known about the epidemiology in infected Swiss herds and risk factors important for transmission in smaller herds. Therefore, the presence of known factors which might favor the spread of paratuberculosis and could be related to the prevalence at animal level of fecal shedding of *Mycobacterium avium* subsp. *paratuberculosis* were assessed in 17 infected herds (10 dairy, 7 beef). Additionally, the level of knowledge of herd managers about the disease was assessed. In a case–control study with 4 matched negative control herds per infected herd, the association of potential risk factors with the infection status of the herd was investigated.

**Results:**

Exposure of the young stock to feces of older animals was frequently observed in infected and in control herds. The farmers’ knowledge about paratuberculosis was very limited, even in infected herds. An overall prevalence at animal level of fecal shedding of *Mycobacterium avium* subsp. *paratuberculosis* of 6.1% was found in infected herds, whereby shedders younger than 2 years of age were found in 46.2% of the herds where the young stock was available for testing. Several factors related to contamination of the heifer area with cows’ feces and the management of the calving area were found to be significantly associated with the within-herd prevalence. Animal purchase was associated with a positive herd infection status (OR = 7.25, p = 0.004).

**Conclusions:**

Numerous risk factors favoring the spread of *Mycobacterium avium* subsp. *paratuberculosis* from adult animals to the young stock were observed in infected Swiss dairy and beef herds, which may be amenable to improvement in order to control the disease. Important factors were contamination of the heifer and the calving area, which were associated with higher within-herd prevalence of fecal shedding. The awareness of farmers of paratuberculosis was very low, even in infected herds. Animal purchase in a herd was significantly associated with the probability of a herd to be infected and is thus the most important factor for the control of the spread of disease between farms.

## Background

Paratuberculosis (PTB) or Johne’s disease is a chronic enteritis of ruminants caused by an infection with *Mycobacterium avium* ssp. *paratuberculosis* (MAP). Clinical signs include profuse diarrhea unresponsive to treatment and significant weight loss in spite of good appetite. Once these symptoms are established, the disease is characterized by chronic wasting with a fatal outcome. Paratuberculosis results in substantial economic losses through decreased production [1], increased replacement rates, and loss of cull value [2].

Infection with MAP typically occurs during the first year of life because calves are more susceptible to infection than older animals [3]. However, clinical signs usually appear only several years after infection, considerably later than the onset of fecal shedding of the germ. During the phase of subclinical shedding, other young cattle may be infected. The pathogen is primarily transmitted by the fecal-oral route [4], but it may also be secreted directly into colostrum and milk [5]; intrauterine transmission has also been described [6].

As no effective treatment exists against PTB, efforts are focused on preventive strategies. Several studies have investigated the control of PTB in affected dairy farms [7-12], but only a few in beef farms [13,14]. Both test-and-cull strategies and strategies of management improvement have been used in such control programs. Most of the studies have been conducted in countries with predominantly large farms housing herds of more than a hundred to over a thousand animals. In contrast, Swiss farms are generally small with an average herd size of 20.3 cows (21.0 in dairy herds and 16.1 in beef herds in 2011; personal communication, Swiss Federal Statistical Office, 2012). As a consequence, management practices also differ from other countries with larger herds.

Little is known about the MAP infection status of cattle herds in Switzerland. In particular, there are no recent studies which describe the prevalence of the disease in the Swiss cattle population or within infected herds. Within-herd seroprevalences were estimated between 4.2% and 21.5% in the early 1990s [15]. Another serological study published in 1997 found that 8% of randomly tested Swiss dairy herds and 0.7% of the tested cattle were serologically positive [16]. Two other studies analyzed bulk-tank milk samples by an IS900 PCR and stated a herd level prevalence of 22.4% [17] and 19.7% [18], respectively. However, the reliability of these results was later questioned as it was recognized that the p90 and p91 primers used in many MAP studies at that time were not as specific as had been assumed [19,20].

The aim of the present study was to acquire further knowledge on MAP infection in affected Swiss dairy and beef herds, to assess the level of disease awareness of herd managers, to identify risk factors associated with high within-herd prevalence, and to identify risk factors which are more common in infected herds than in herds without a PTB history.

## Methods

### Recruitment of infected herds

Twelve infected dairy and nine infected beef herds were identified based on the records of the Ruminant Clinics of the Vetsuisse Faculty in Berne and Zurich, of veterinary practitioners and of state veterinary officers. The farmers were then invited to join the study by phone call. The main inclusion criterion was the presence of at least one confirmed case (by means of serology, Ziehl-Neelsen staining, PCR and/or culture) of clinical PTB during the five years preceding the start of the study. The confirmed cases had to have been born on the farm.

### Herd visits and sampling procedures

The 21 herds included in the study were visited between February 2011 and July 2011. During these visits, the husbandry practices by age group, the feed storage, water supply, and manure storage were recorded. Furthermore, a questionnaire was completed through an interview with the farmers to assess potential risk factors for PTB on the farm, with different questionnaires for dairy and beef farms. Both questionnaires had previously been evaluated on test farms for applicability and clarity before they were used on the study farms. They included open and closed questions related to general characteristics of the farms, such as size, cattle breed(s) and production level, as well as targeted questions referring to potential risk factors for MAP transmission, chosen based on available information from the literature [21,22], and questions about the previous PTB history of the farms. The knowledge of the herd managers about PTB was also assessed by means of the questionnaires. The original questionnaires are available from the corresponding author upon request.

A fecal sample of each animal of one year of age or older was collected for MAP testing. Cattle that had been bought from other farms only for fattening and were kept separately from the herds were excluded from the sampling. Obstetric gloves used for fecal sample collection were changed between animals. The samples were sent to the laboratory overnight and reached it within 24 hours. After arrival, they were frozen at -20°C if immediate processing was not possible. The duration between sampling and processing ranged from one day to 3.3 months.

### Laboratory analyses

Fecal samples from three animals of the same herd (2 g/animal) were pooled for MAP analysis. The pools were analyzed according to established methods, both by culture [23] and F57 RT-PCR [24]. For pools that tested positive in either method, the culture and PCR of the corresponding individuals were repeated, using the frozen fecal samples from which the pools had been formed. If pools were positive but the consecutive individual samples were negative, the respective animals were tested again, using PCR of a fresh fecal sample and serology (ELISA; IDEXX Paratuberculosis Screening Ab Test, IDEXX Montpellier SAS, France).

### Control herds

Four control herds per infected herd were enrolled to evaluate whether classical risk factors for MAP transmission were more often present on infected farms than on PTB-free farms. These herds had to be without any history of PTB for the ten years preceding the start of the study. They were matched to the individual infected herds by type (dairy/beef), size, cattle breed and region. The questionnaires used for the visits of the control farms were similar to the questionnaires for the infected farms, except for the questions about the PTB history of the herd which were deleted, as well as some general questions about the farms not directly related to PTB transmission, as personnel, forage, disease prevention, herd health issues, and future of the farm.

### Statistical analyses

The study was designed as a case–control study for the identification of risk factors in the management of infected farms as compared with control herds, while risk factors associated with high prevalence levels were evaluated in relation to the MAP excretion levels within infected herds.

For comparison of infected herds with control herds, univariable analysis using logistic regression was applied as an initial screening test to identify potential risk factors associated with the herds’ infection status. This was followed by conditional logistic regression that included all variables with a p-value of < 0.2 in the univariable analysis. Three analyses were performed, first for the factors that were present in both dairy and beef herds, and subsequently for potential risk factors associated with infection in dairy or beef herds separately. Competing models were analyzed using the Akaike Information Criterion (AIC) and the best model was selected on the basis of the lowest AIC**.** The significance level was set at p < 0.05. Because the control herds had not been confirmed as truly negative other than based on history, a second analysis was undertaken with the assumption that 10% (the assumed herd prevalence of Switzerland) could still be positive in reality. Briefly, a Monte-Carlo simulation was carried out. Each of the control herds was randomly assigned a disease-positive status with a probability of 10%. The conditional logistic regression was repeated using the modified data set and a new set of modified parameters was estimated. This was repeated 10,000 times to build up a probability distribution of the modified parameters in the model and statistical inference was calculated on this probability distribution. Thus there were 10,000 replicates of the parameters and from these 10,000 replicates the median and 95 percentiles could be calculated.

Further analyses were performed to investigate the relation between potential risk factors and the prevalence of MAP shedding within infected herds using logistic regression. However, in this case, only a univariable analysis was conducted as multivariable analysis was not possible due to the small number of infected herds participating in the study. Likewise, three analyses were performed: one for all herds, one for dairy and one for beef herds separately. For calculation of the within-herd prevalence, the infection status of animals aged two years or older only was considered, in order to have a comparable sample population across all farms, as the heifers younger than two years of several herds were kept on separate farms under rearing contract and therefore were not available for testing.

This study is reported in conformity with the STROBE statement for reporting observational studies [25]. All statistical analyses were undertaken in R [26].

The study was conducted in accordance with the animal welfare legislation of Switzerland, all (including ethical) aspects of the study had been previously approved by the Swiss Veterinary Office. All farmers were thoroughly informed about the project prior to the herd visit and gave their informed consent for the sampling of fecal specimens from their animals and for completion of a questionnaire regarding herd management practices.

## Results

### Situation in PTB infected Swiss dairy and beef herds

Twelve dairy herds and nine beef herds were enrolled in the study between January and July 2011. Two dairy and two beef herds were later excluded from further analyses because all fecal cultures were negative for MAP. The four excluded herds were small with 11 to 21 tested animals.

A total of 1120 animals older than one year were tested for MAP shedding in the 17 remaining herds. The number of tested animals per herd ranged from 25 to 130 (33 to 130 for dairy herds, 25 to 92 for beef herds), with an average of 66 animals older than one year (73 for dairy and 56 for beef herds) or 49 cows (55 for dairy and 41 for beef herds), respectively. Several breeds were represented in the infected herds. The dairy herds included three Red Holstein herds, three Holstein Friesian herds, one Jersey herd, one mixed herd with Red Holstein and Swiss Fleckvieh, one mixed herd with Holstein Friesian and Brown Swiss, and one Brown Swiss herd with some Jerseys. In the latter herd, PTB cases had been recorded in the Jersey population only. The beef cattle population under study consisted of four Limousin herds, two Angus herds and one Charolais herd.

The most important data about general management practices are listed in Table 1. The heifers of most farms that were sent to mountain pastures shared these pastures with heifers from other farms (86%) and this was also the case for 50% of the mountain pasturing beef. Of the 13 farms that purchased cattle, 24% purchased one animal per year on average, and 53% bought two to five animals per year.

**Table 1:** Management practices in 17 infected farms

**Variables (number of farms dairy/beef)**	**Number of farms (%)**	
	**Dairy**	**Beef**
General management practices		
Stall system (10/7)		
Free stall barn	8 (80)	7 (100)
Tethered stable	2 (20)	0 (0)
Summer mountain pastures (10/7)		
Heifers	7 (70)	0 (0)
Heifers and cows	1 (10)	0 (0)
Whole herd (calves included)	1 (10)	4 (57)
No purchase^1^ (10/7)	1 (10)	1 (14)
Calving facilities and management		
Type of calving pen (10/7)		
Individual calving pen	5 (50)	5 (71)
Group calving pen	3 (30)	1 (14)
No calving pen	2 (20)	1 (14)
Calvings in the barn (8/6)		
10-50%	2 (25)	2 (33)
>50%	0 (0)	1 (17)
Cleaning of calving pen (8/6)		
Change of bedding after each calving	1 (13)	0 (0)
Possibilities of contamination from cows to calves or heifers		
Preweaned calves (10)		
Kept in same building as cows	4 (40)	N/A
Direct contamination of calf area with cow manure	5 (50)	N/A
Direct contamination of feed with cow manure	1 (10)	N/A
Direct contamination of water supply with cow manure	2 (20)	N/A
Weaned calves (10)		
Kept in same building as cows	3 (30)	N/A
Direct contamination of calf area with cow manure	3 (30)	N/A
Direct contamination of feed with cow manure	1 (10)	N/A
Direct contamination of water supply with cow manure	0 (0)	N/A
Heifers >1 year (10/7)		
Kept in same building as cows	4 (40)	5 (71)
Direct contamination of heifer area with cow manure	2 (20)	5 (71)
Direct contamination of feed with cow manure	0 (0)	1 (14)
Direct contamination of water supply with cow manure	1 (10)	1 (14)

^1^No purchase: Herds with no purchase were closed herds that bought no animals at all.

N/A: not applicable.

Calving of over 90% of the cows in individual calving pens was observed in only three farms (18%). In the other farms, it could happen that two cows shared the same calving pen, that they had group calving pens, or that calvings took place in the barn (Table 1).

In the dairy herds, calves were separated from their dams within one hour after birth in 80% of the herds, while they stayed with their dams during their first day of life in the other 20%. However, it could not be excluded that the calves had an opportunity to suckle their dam before being separated in 70% of the herds. Most of the calves (in 80% of the dairy farms) received colostrum from their own dam only. In the remaining 20%, calves from first-calf heifers occasionally received colostrum from an older cow. After the colostrum, all were fed bulk tank milk, except on one farm where they were fed a milk replacer. Waste milk containing antibiotics or high somatic cell counts was also fed in 78% of the farms feeding milk.

In the beef herds, the calves usually stayed in the herd with their dams for ten months. During that time, a straw-bedded area to which the adult did not have access was available for the calves in all farms.

In 40% of the dairy farms, the young stock went to contract heifer-rearing facilities at an age between one and four months. In contrast, only one beef farmer sent roughly 10% of his heifers at the age of 14 months to another farm. Dairy and beef heifers generally stayed at the contract rearing farm until approximately one month before calving.

During the summer, 20% of young dairy cattle aged less than a year and 71% of all (dairy and beef) heifers aged more than a year were kept on pastures that were either pastured by cows or where slurry from the cow herd had been spread during the same season. In all beef herds, with one exception, slurry was also spread during the pasturing season.

### Farmers’ knowledge about PTB in infected and control herds

In general, the farmers’ knowledge about PTB was limited on infected farms and very low on control farms (Table 2). Little was known by the managers of infected farms about preventive strategies: less than a third of them (30% dairy and 29% beef) were aware of the importance of good calving management practices, 40% of the dairy farmers knew that separating calves from their dams immediately after birth was important and 40% knew that calves should not suckle their dam, but only 10% were aware that every contact between cows and calves should be avoided.

**Table 2:** Farmers’ knowledge about paratuberculosis in 17 infected and 68 control herds

**Variables**	**% of farmers**			
	**Infected herds**		**Control herds**	
	**Dairy**	**Beef**	**Dairy**	**Beef**
Existence of PTB	100	100	40	32
Symptoms				
Diarrhea	100	100	25	14
Emaciation	100	100	15	18
Reduction in milk yield	80	43	8	11
Epidemiology				
Infection mainly in first year of life	40	43	3	11
Long incubation time	80	86	8	11
Infection through feces	90	86	3	4
Infection through milk	80	43	0	4
Intrauterine infection	30	14	0	4

### Prevalence of MAP excretion

In total, 1120 animals were tested for MAP excretion. As the young stock was kept on rearing contract farms in some of the herds, only the 913 animals (602 from dairy herds and 311 from beef herds) aged two years or older were considered for the determination of prevalence in order to have a valid comparison across all herds. Of the animals aged two years or older, 46 (5.0%) were positive based on the results of individual cultures and 11 (1.2%) were positive in individual PCR. In addition, samples of 10 pools that had been positive in the culture gave negative results in individual culture. Two positive animals in such pools could be identified later, one by serology and one by PCR of a fresh fecal sample. For the 8 other pools, the presence of one positive animal in each positive pool was assumed for further calculations. With these additional animals, the total number of positive animals was determined to be 56, which corresponds to an overall prevalence of 6.1% (6.0% dairy and 6.4% beef cattle). Of the animals between one and two years of age not included for further analyses, eight animals (3.9%) from 6 farms (out of 13 where the young stock was available for testing) were positive in the culture, resulting in a herd prevalence of 46.2%, whereas none was positive in the PCR. The results of culture and PCR and the characteristics of the herds with their sizes and prevalence rates are listed in Table 3.

**Table 3:** **Results of culture and PCR for *Mycobacterium avium *subsp. *paratuberculosis *in 17 infected herds**

**Herd**	**Production type**	**Herd size (all cattle > 2 years)**	**Number of animals < 2 years**	**Prevalence of PCR positive animals > 2 years (%)**	**Prevalence of culture positive animals > 2 years (%)**	**Prevalence of culture positive animals < 2 years (%)**
Total (mean)	Dairy	60.2	12.5	0.7	6.0	3.2
Total (mean)	Beef	44.4	11.7	2.9	6.4	4.9
1	Dairy	82	6	0	1.2	0.0
2	Dairy	53	0	0	1.9	N/A^1^
3	Dairy	28	5	0	3.6	0.0
4	Dairy	66	22	0	4.5	9.1
5	Dairy	39	10	2.6	5.1	0.0
6	Dairy	57	1	0	5.3	N/A^1^
7	Dairy	48	0	0	6.3	N/A^1^
8	Dairy	55	15	1.8	7.3	0.0
9	Dairy	81	29	1.2	7.4	3.4
10	Dairy	93	37	1.1	12.9	2.7
11	Beef	54	12	1.9	1.9	0.0
12	Beef	41	7	0	2.4	0.0
13	Beef	56	21	0	3.6	4.8
14	Beef	28	12	3.6	7.1	8.3
15	Beef	77	15	2.6	7.8	0.0
16	Beef	31	14	0	12.9	14.3
17	Beef	24	1	12.5	16.7	N/A^1^

^1^ N/A = not applicable; no prevalence could be calculated for farms 2 and 7 with no animals < 2 years and for farms 6 and 17 with only one animal < 2 years at the time of the farm visit.

Because culture sensitivity was higher compared to PCR and because all PCR positive animals were also positive in the culture, only culture results were used for statistical analyses. Detailed results on the performance of culture and PCR will be published elsewhere.

### Risk factor analysis

Table 4 shows the results obtained with the final model for statistical comparison of risk factors present in infected herds versus control herds. The risk factor “animal purchase” was associated with a positive herd infection status in two categories (all herds and dairy). The odds ratio (OR) for this factor in the beef group was not significant (OR = 9.19, p = 0.061, 95% confidence interval CI 0.90-93.51). In the Monte-Carlo analysis “animal purchase” remained significant in the analysis of all herds (OR = 5.15, p = 0.025, 95% CI 1.33-20.13).

**Table 4:** Risk factors associated with a positive infection status (comparison of infected herds vs. control herds)

**Variables**	**% cases/% controls**	**OR**^**1**^	**95% CI**^**2**^	**p-value**
All herds				
Animal purchase^3^	53/16	7.25	1.86-28.34	0.004
Dairy herds				
Animal purchase^3^	60/23	6.34	1.18-34.13	0.031
Beef herds				
No significant factors				

^1^OR = odds ratio.

^2^95% CI = 95% confidence interval.

^3^Farms that purchased two or more animals per year on average compared to farms that purchased up to one animal per year on average.

The results of the univariable analysis investigating the relation between the risk factors observed on the farms and the within-herd prevalence in infected herds are shown in Table 5. In the categories all herds and dairy, risk factors regarding the heifer husbandry were associated with higher within-herd prevalence. Several factors regarding the calving hygiene were significantly associated with within-herd prevalence. For the factor “animal purchase”, a significant negative association with prevalence (OR < 1) was found in dairy herds. No significant factors were identified for beef herds.

**Table 5:** Risk factors associated with the prevalence level of MAP excretion within infected herds

**Variables**	**OR**^**1**^	**95% CI**^**2**^	**p-value**
All herds			
Direct contamination of heifer area^3^	1.94	1.12-3-33	0.017
Heifers kept in the same building as cows	1.79	1.02-3.17	0.044
Direct contamination of heifer water supply^4^	3.00	1.61-5.59	0.0005
Calving pen used as sick cow pen	0.47	0.26-0.87	0.017
Dairy herds			
Direct contamination of heifer area^3^	2.40	1.18-4.88	0.016
Direct contamination of heifer water supply^4^	2.99	1.44-6.22	0.003
Heifers kept on contaminated pastures^5^	2.43	1.09-5.43	0.030
Type of calving pen^6^	2.11	1.07-4.15	0.031
Calving pen used as sick cow pen	0.33	0.16-0.71	0.004
Cleaning calving pen with high-pressure washer > 1x/year	2.13	1.07-4.26	0.031
Animal purchase^7^	0.51	0.26-0.99	0.048
Beef herds			
No significant factors			

^1^OR = odds ratio.

^2^95% CI = 95% confidence interval.

^3^Farms where direct contamination of the heifer area by manure spilling from adult animals could happen compared to farms where such contamination was not possible.

^4^Farms where direct contamination of the water supply of the heifers by manure spilling from adult animals could happen compared to farms where such contamination was not possible.

^5^Pastures contaminated through pasturing of cows or slurry spread earlier in season.

^6^No calving pen in free stall or boxes as in free stalls associated with higher prevalence than box with deep straw bedding and calving in tethered stable.

^7^Farms that purchased two or more animals per year on average compared to farms that purchased up to one animal per year on average.

## Discussion

The present study allowed for identifying the management parameters in Swiss cattle herds that are significantly associated with the infection status in regard to PTB (animal purchase) and with the prevalence of fecal MAP excretion within infected herds (mostly factors related to heifer management and to the calving area). Similar within-herd prevalence values were observed in beef and in dairy operations. A further important observation is that the knowledge level of the farmers about PTB was extremely low.

Regarding the infection status of a herd, we discuss mostly the influence of animal purchase, which was the most important factor identified upon comparison of 17 infected herds (10 dairy and 7 beef) with 68 matched uninfected control herds (i.e. 4 control herds per infected herd), as well as shared pastures, herd size and sample size. The discussion of risk factors associated with the within-herd prevalence includes direct and indirect contact of heifers and calves with fecal material from adult animals, as well as management and hygiene in the calving area. Finally, the low level of knowledge of the farmers regarding PTB in infected and control herds and the consequences thereof are addressed at the end of the discussion.

### Risk factors associated with the infection status of the herds

The analysis of management factors in infected herds in comparison to control herds showed that a positive infection status was associated with higher intensity of cattle movement, i.e. with regular purchase and introduction of animals into the herds. Farms that bought more than one animal per year had a higher risk of being infected than farms that bought an average of one animal per year or less. The fact that animal purchase was the only parameter remaining statistically significant in all models used for comparison of infected and control herds suggests an important effect on the PTB infection status of Swiss cattle herds, which is in accordance with reports from other countries [27-30]. In beef farms that bought up to one animal per year, the breeding bulls changed every 2–3 years were often the only purchase at all. Only 2 herds (11.8%) were completely closed and did not purchase any animals, while 53% of all infected farms purchased more than one animal per year. The percentage of control farms purchasing more than one animal per year was significantly lower (16%, p = 0.005). This suggests that the introduction of new animals in the herd beyond the absolute minimum increases the risk of MAP infection. This is in accordance with the well-established recommendation not to buy cattle from sources of unknown PTB status in order to avoid introducing the disease into uninfected herds [12,29]. These results should be interpreted with caution because selection of the control herds was based on matching by herd type, size, breed and localization, under the condition that they had had no animals showing suspect signs of PTB during the 10 years prior to the study, as confirmed by the farmers. The disease-free status of the control herds, however, was not confirmed e.g. by fecal culture. This potential bias was further evaluated by means of Monte-Carlo analysis and the result for animal purchase remained significant for all herds (p = 0.025). The fact that it was no longer significant if dairy and beef herds were analyzed separately is likely due to the resulting smaller sample size in each of the herd type groups in combination with the fact that, due to the four times greater number of control herds than infected herds, almost 30% of the herds considered to be infected were, in fact, herds with no real evidence of PTB according to the model’s assumption of 10% “infected” control herds.

One feature particular to Switzerland and the surrounding alpine countries is the common pasturing of cattle from several herds on mountain pastures. It is especially common for heifers to graze on high pastures in remote areas. Common pasturing of animals from several herds could potentially become a source of infection for negative herds, particularly for beef cow herds as the calves share their pasture with the adult cows; indeed, 2 of the beef herds (29%) shared mountain pastures with at least one other herd during the summer. A potential risk of infection may also exist for the 60% of dairy heifers which shared their summer pastures with heifers from other herds. A significant effect of shared pastures on the herd infection status was, however, not observed.

The present results did not allow determining if the absence of other significant risk factors regarding the infection status of the herds under study was due to the limited sample size or to the fact that management practices are fairly uniform in Switzerland. Indeed, only very few differences were observed between the infected and the control herds.

The average size of the infected herds participating in the study (49 cows on average, 55 in dairy and 41 in beef herds, respectively) was more than twice as big as the average herd size in Switzerland (20.3 cows, 21.0 in dairy and 16.1 in beef herds in 2011; personal communication Swiss Federal Statistical Office, 2012). Although this observation must be interpreted with caution because the herds included in the study were not necessarily representative of the entire population of herds affected with PTB in Switzerland, it is in accordance with studies conducted elsewhere [28,29]. Whether a larger herd size leads to increased awareness of the disease, as a higher number of animals become clinically ill each year, or whether management practices in use in larger herds vs. small herds lead to a higher MAP transmission rate, or whether the above average size of the infected herds included in the study was due to other reasons, could not be determined in the presented study. A bigger sample size might have allowed a better insight into this question, as well as higher statistical power in general, but it was not possible to include more herds due to practical constraints related to laboratory capacity for the time-consuming fecal cultures for MAP, despite the fact that three fecal samples were pooled for analysis as described previously [31,32] in order to avoid unnecessary laboratory expenditure. However, the management practice identified as being associated with an elevated risk of MAP infection in this relatively small sample, i.e. animal purchase, is likely to be a truly important factor influencing the epidemiology of PTB in the Swiss dairy and beef cattle population.

### Risk factors associated with the within-herd prevalence of MAP shedding

Although the pooling of three samples for culture and PCR might have led to a slight underestimation of the true prevalence in the infected herds, the observed prevalence values were generally low, with a mean within-herd prevalence of 6.0% in dairy and 6.4% in beef herds, respectively. While only 1 dairy herd out of 10 had a prevalence above 10%, a prevalence higher than 10% was observed in 2 out of 7 beef herds (28.6%). This is in contrast to other reports of lower prevalence values in beef than in dairy herds in North America [14,33]. This might be due to the intensive management of beef herds in Switzerland associated with limited availability of pasturing surfaces.

Higher within-herd prevalence was primarily associated with potential for contamination of the heifer area with feces from adult animals (housing in the same building, direct contamination of the heifer area and of the water supply, and pasturing of the heifers on surfaces where the cows had pastured or where slurry from the cows had been spread during the same season). This is in contrast to the results of a systematic review of literature regarding risk factors for MAP transmission [4] which showed the most important parameter to be contact of calves with adult cow feces. According to the results of the present study, this parameter appears to be relevant also for heifers. While direct contamination of the heifer area with cow manure was possible in 20 (dairy) to 71% (beef) of the herds and potential direct contamination of the water supply was present in 10 (dairy) to 14% (beef) of the farms, all heifers -except for one farm- were allowed to graze on pastures that could have been contaminated by adult stock. Fecal shedding of MAP in very young animals (<2 years) was observed in 3 beef and 3 dairy herds, whereby in both groups a single shedder was observed in 2 herds and 2 shedders in one herd. Considering that the heifers of several herds were away on rearing contracts at the time of fecal sampling, the proportion of herds with shedding young animals was similar in both groups, with 50% (3/6) in beef herds and 42.9% (3/7) in dairy herds. The role of the exposure of heifers to MAP in the transmission cycle should be further evaluated in the light of the unexpected high number of animals younger than 2 years shedding MAP and of the fact that 40% of the infected herds send their heifers to contract rearing farms, where they could potentially infect heifers from other farms.

The level of significance was not reached for the same contamination pathways for younger calves, although it is generally admitted that calves play a greater role in the transmission cycle of MAP [3]. In the present study, potential contamination from cows to calves was only analyzed for the dairy herds (i.e. on a low number of herds), as cows and calves were kept together for approximately 10 months in all beef herds. In dairy herds, the calves were often kept in close contact to the cow herd or at least in the same building, and numerous opportunities for contamination of the young stock areas, including water and feed, with manure from adult animals were observed. Indeed, contamination of the calves with cows’ feces was possible at one time or another in most of the infected dairies (70%). Since contamination was assessed on a binomial basis (yes/no) only, the severity and/or duration of the contamination was not included in the analyses. The only factor significantly associated with higher within-herd prevalence of MAP shedding in regard to the calves was the type of calving pen in dairy herds. Farms where the cows calved in straw-bedded boxes or in tethered stables had lower prevalence values than farms where cows calved in the barn or in a separated area with several free stall boxes, potentially exposing the newborn calves to the feces of several cows. In Swiss farms, calving pens are normally used for only one or two cows at a time, and not for larger groups. However, lack of hygiene in the calving area and less than optimal calving management practices were frequently observed in infected farms, e.g. the birth of calves in the barn instead of the calving pen or the sole addition of fresh bedding instead of emptying and cleaning the calving boxes between calvings. As the calving area is considered to be a high risk area for transmission of MAP to newborn calves [8,28,29], recommendations for the control of PTB should always include measures aiming at improved hygiene in the calving area and rapid removal of the newborn dairy calves from their dams [34]. Thus, although the dairy calves were generally separated from their dams within one hour of their birth, a need for improved hygiene measures especially regarding the calving management was identified in the infected herds under study.

Shedding of MAP into the colostrum and milk of infected cows has been described as a risk factor for infection of young calves [5]. In the present study, dairy calves were generally fed the colostrum of their own dam only. Pooling of colostrum, which has been described as a risk factor for the transmission of MAP [35], was not observed in any herd included in the study. On the other hand, the use of milk replacer or pasteurized milk to feed calves is not a common practice in Swiss dairy herds and was observed in one single dairy herd. However, based on the low within-herd prevalence observed in most dairy herds and because milk has been reported to be of limited importance for MAP transmission [4], this point may be a minor issue.

### Awareness of the farmers about PTB

In general, the knowledge level about PTB of the herd managers was extremely low, especially in the control farms. Almost two thirds of the managers of control herds stated that they had never heard of the disease. This suggests that PTB is not an important issue for Swiss farmers. The level of knowledge about the disease was also low in infected farms. While 100% of the herd managers knew the cardinal symptoms of diarrhea and emaciation, only 80% (dairy) and 43% (beef) farmers were aware of the reduction in milk yield associated with MAP infection. The epidemiology of the disease, especially the infection pathways that can be influenced by preventive strategies were understood by only 14% (intrauterine infection, beef) to 90% (infection through feces, dairy) of the managers in infected herds, i.e. farmers who had already had at least one confirmed clinical case of the disease in their farm. This indicates that increased efforts aimed at raising the awareness of PTB of the Swiss veterinarians are necessary in order to improve the transfer of this knowledge to the farmers.

Some results of the risk factor analysis within infected herds suggest that control measures were more likely to have been implemented in herds with high prevalence of MAP shedding. Indeed, cleaning the calving pen with a high-pressure washer > 1x/year was surprisingly associated with a higher MAP prevalence in the analysis of the dairy herds. This can likely be explained by the higher awareness of farmers having suffered the greatest financial losses due to PTB about the importance of hygiene, especially in the calving pens. This was confirmed by the farmers’ answers to the questionnaire (data not shown). The “protective” OR calculated for increased intensity of animal purchase in dairy herds can likely be explained by the same reason, beside the fact that this factor is rather associated with the herd status (infected or not) than with the within-herd prevalence. Likewise, using the calving pen as a sick cow pen appeared to reduce the risk of MAP transmission, which is also rather associated with the increased awareness of farmers suffering a higher prevalence of the disease. Indeed, the two herds with the highest prevalence of MAP excretion in their category (dairy herd 10 with 12.9% and beef herd 17 with 16.7%, respectively) had separated pens for calvings and sick cows in their barns, which might explain this unexpected result due to the large influence of single herds given the small sample size in the present study.

## Conclusions

Despite the limited number of herds included, this study allows for a first description and evaluation of the situation in MAP infected herds under traditional farming conditions in smaller farms, i.e. in an agricultural system different from those mostly described in the literature from North America or Australia. While the presence of classical risk factors for PTB associated e.g. with calving management practices was confirmed in small infected cattle herds, unexpectedly numerous significant risk factors associated with the management of heifers older than one year were identified. This underlines the importance of avoiding contamination with feces from adult animals not only for young calves but also for older breeding stock. Furthermore, the fact that animal purchase has been shown to be the most significant factor determining the infection status of a herd, this parameter should be taken into consideration for the development of programs designed to control the spread of PTB among herds in the future. Based on the limited knowledge of farmers about PTB observed in the present study, increased information efforts toward Swiss herd managers and veterinarians are needed to raise their level of awareness about the disease and its control.

## Abbreviations

PTB: Paratuberculosis; MAP: *Mycobacterium avium* subsp. *paratuberculosis*; AIC: Akaike Information Criterion; OR: Odds ratio; CI: Confidence Interval.

## Competing interests

The authors declare that they have no competing interests.

## Authors’ contributions

RK coordinated the study, conducted the farm visits, analyzed the data and drafted the manuscript; PT planned and performed the statistical analyses; SK performed the MAP cultures and PCR; MW and RS participated in the planification of the study and supervised the laboratory work; GKB participated in the conception of the study and the redaction of the manuscript; BB participated to the farm visits and to the drafting of the manuscript; MM conceived and coordinated the study, helped to draft and finalized the manuscript; All authors have read and approved the manuscript.
